# Neuroanatomy of spinal nociception and pain in dogs and cats: a practical review for the veterinary clinician

**DOI:** 10.3389/fvets.2025.1534685

**Published:** 2025-02-20

**Authors:** Tenna Remler Pedersen, Mette Berendt, Clare Rusbridge

**Affiliations:** ^1^Department of Veterinary Clinical Sciences, Faculty of Health and Medical Sciences, University of Copenhagen, Frederiksberg, Denmark; ^2^Department of Veterinary Clinical Sciences, School of Veterinary Medicine, University of Surrey, Guildford, United Kingdom

**Keywords:** chronic pain, maladaptive pain, nociceptive pathways, periaqueductal gray, Rexed laminae, spinocervicothalamic tract, spinothalamic tract, thalamus

## Abstract

Chronic pain is a prevalent condition in companion animals and poses significant welfare challenges. To address these concerns effectively, veterinary clinicians must have a comprehensive understanding of the neuroanatomy of nociception and the intricate processes underlying pain perception. This knowledge is essential for planning and implementing targeted treatment strategies. However, much of the existing information on pain mechanisms is derived from studies on rodents or humans, highlighting the need for further translational research to bridge this gap for veterinary applications. This review aims to provide veterinary clinicians with an in-depth overview of the spinal nociceptive pathways in the dog and cat, tracing the journey from nociceptor activation to cortical processing in the brain. Additionally, the review explores factors influencing nociceptive signaling and pain perception. By enhancing the understanding of these fundamental physiological processes, this work seeks to lay the groundwork for developing effective therapies to manage the complexities of chronic pain in companion animals.

## Introduction

Effective pain management is a critical aspect of companion animal care. However, translating findings from rodent-based pain research into practical clinical strategies for veterinary medicine remains a significant challenge. The veterinary literature has historically focused more on the assessment and management of acute pain, with chronic pain—apart from osteoarthritis-related conditions—receiving comparatively less attention. This gap leaves veterinary surgeons less equipped to objectively monitor and manage chronic pain conditions in their patients.

Understanding the mechanisms of pain generation and the maladaptive processes that characterize chronic pain is a crucial step in developing effective multimodal treatment approaches. This review aims to provide veterinary clinicians with a comprehensive overview of the neuroanatomy of feline and canine spinal pain pathways. It covers the journey from nociceptor activation through the spinal cord dorsal horn, ascending spinal tracts, and relays to the somatosensory cortex. Additionally, it explores factors that influence pain perception, including spinal and supraspinal modulation, cognitive and emotional influences, and anatomical differences between dogs and cats. By integrating this knowledge, clinicians can improve their ability to manage chronic pain in companion animals effectively.

## What is pain?

The International Association for the Study of Pain (IASP) defines pain as “*an unpleasant sensory and emotional experience*,” implying that pain is not just a matter of sensation but also a conscious registration and emotional consequence (1). This makes pain a very complex sensation and experience with wide individual variety. In veterinary medicine, there is no veterinary-specific definition of pain; however, the IASP definition of pain states that “inability to communicate does not negate the possibility that a human or a nonhuman animal experiences pain” (1). There is therefore a common understanding that the definition by IASP applies to animals as well as humans.

Pain perception and the nociceptive system are integrated parts of the nervous system that allows an individual to interact with the environment and react accordingly to the received stimuli. In most cases, the purpose of feeling pain is to protect the individual from further harm and facilitate healing (2).

It is important to distinguish between “pain” and “nociception” (coming from the Latin word *nocére*, meaning to injure or harm). *Pain* encompasses a subjective and conscious perception whereas *nociception* refers to the neuronal mechanisms that encode noxious stimuli (1). These two terms are often used interchangeably, as the two phenomena often occur together. However, it is possible to have nociception without pain, as in the case of general anesthesia during surgery (2). Although the body registers and processes noxious stimuli from the surgeon’s scalpel, the patient does not consciously perceive the pain. Also, it is possible to have pain without nociception, as in the case of phantom pain described in cats after limb amputation (3).

*Nociceptive* pain arises from actual or threatened damage to non-neural tissue due to the activation of specialized receptors (nociceptors) in the peripheral nervous system (4). These receptors respond to one or more types of noxious stimuli of mechanical, thermal, or chemical (e.g., inflammatory) origin. Nociceptive pain is the most common type of acute pain but may become chronic (2). Acute pain is often *adaptive* in nature, meaning it ceases to exist once healing is completed, whereas chronic pain is characterized by becoming *maladaptive* because it persists longer than expected (2). Currently, there is no consensus on when pain is considered chronic, but in humans it is commonly accepted that pain that exists for longer than expected for a given injury, or for more than 3 months is considered chronic (2). It has been suggested that this definition should be adapted and shortened for companion animals to reflect their shorter lifespan (5). *Neuropathic* pain is not caused by activation of nociceptors but is due to damage to or disease of the somatosensory nervous system. *Nociplastic* pain is proposed as a term to explain evoked types of pain, in which the patient experiences pain without any apparent reason due to alterations in the somatosensory system (4). Nociplastic pain has been shown to exist in humans and rodent models (6–8). While strong evidence for nociplastic pain in dogs or cats is currently lacking, it is anticipated that this type of pain occurs in these species as well.

### From nociception to pain perception

**Figure 1:**
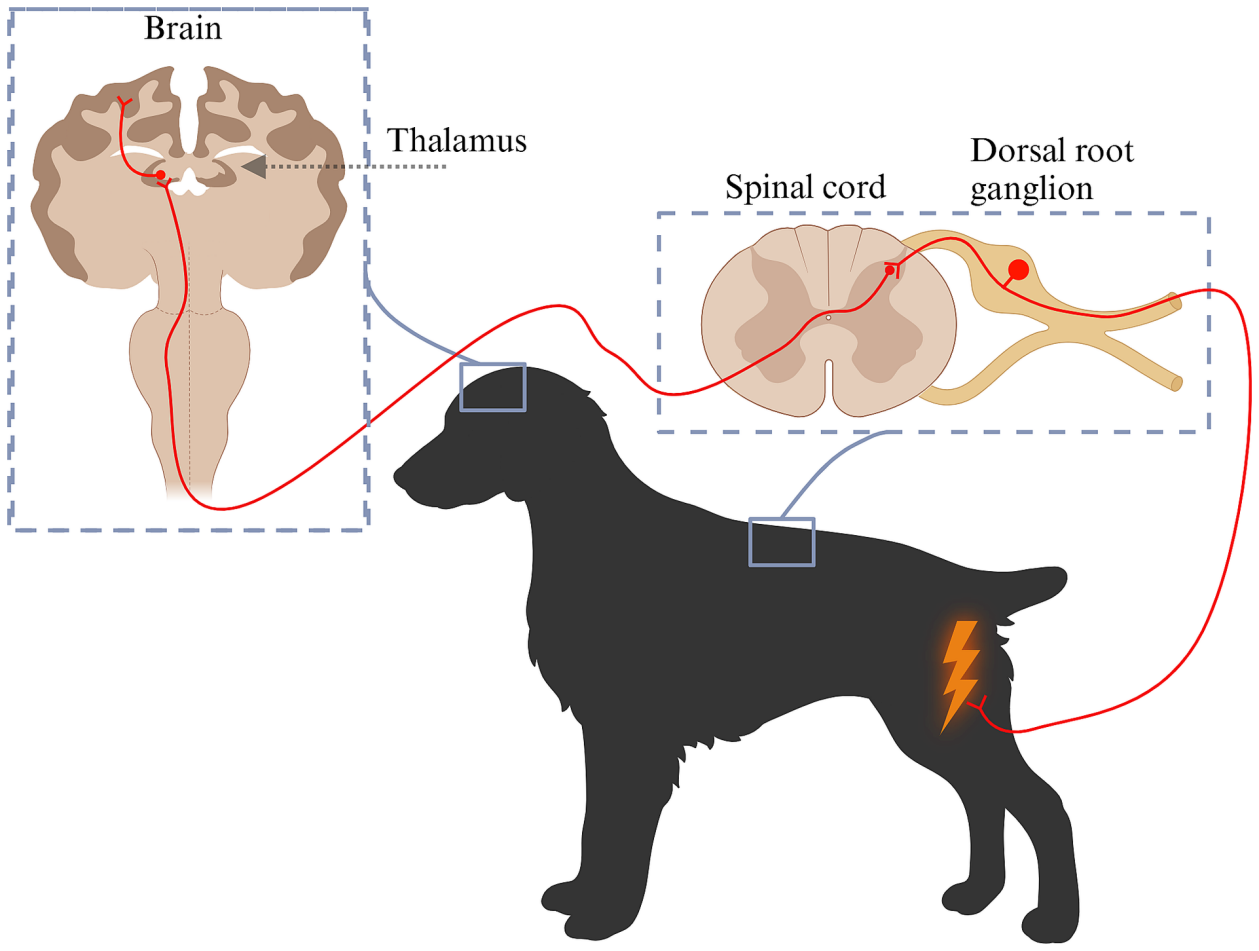
Schematic diagram of pain pathways from nociception to pain perception. Pain is initiated by specialized nerve receptors known as nociceptors. Nociceptors become activated by various types of stimuli: mechanical stimuli (cutting, pressure), temperature or chemical changes (e.g., inflammation). The generated signal is transmitted to the neuronal cell body located in the dorsal root ganglion, from where it is relayed to the dorsal horn. Within the dorsal horn, the primary neuron synapses with a projection neuron or an interneuron that works to modulate incoming inputs by either enhancing or reducing them. Projection neurons transmit signals to the brain including the thalamus, from where they are distributed onwards to different brain structures and perceived as pain. Created in BioRender. Pedersen (2025) https://BioRender.com/y80d045.

In simple terms, nociceptive pain is initiated by activation of specialized nerve receptors located at the end of axons from nerves originating from the dorsal root ganglion (9, 10). These receptors, known as nociceptors, detect harmful stimuli affecting peripheral tissues and generate electrical signals that are transmitted through the nervous system (Figure 1). Nociceptors are broadly classified based on their responsiveness to various stimuli and may be activated by one or multiple types of stimuli (11). Mechanosensitive nociceptors primarily respond to mechanical stimuli, such as cutting or pressure, while thermal and chemical nociceptors are activated by changes in temperature or chemical signals. Mechanothermal nociceptors are responsive to both mechanical and thermal stimuli, whereas polymodal nociceptors exhibit broad functional diversity, detecting and responding to a combination of mechanical, thermal, and chemical stimuli (11). Nociceptors can be further classified as high- or low-threshold nociceptors based on the intensity of stimuli required to activate them (12). Low-threshold mechanoreceptors (LTMRs) respond to low-intensity mechanical stimuli such as touch, while high-threshold mechanoreceptors (HTMRs) are specialized to detect potentially harmful mechanical stimulation (12). Nociceptive information is transmitted from nociceptors to neurons in the dorsal root ganglion and then directly into the dorsal horn of the closest spinal cord segment or via Lissauer’s tract to an adjacent spinal cord segment (13). Approximately 80% of the axons travelling through the Lissauer’s tract are unmyelinated fibers (13). In the spinal cord, the neuron from the dorsal root ganglion synapses with either a projection neuron or an interneuron. Interneurons are abundant in the spinal cord and help modulate incoming inputs by either enhancing or reducing them (14). This helps to control the magnitude of the inputs that are projected to the brain. In contrast, projection neurons transmit signals to the brain thereby participating in generating a response to pain (15). Depending on where they terminate in the brain, the response may be either conscious or subconscious (16, 17). The brain can modulate inputs by sending signals to the spinal cord, which can either amplify or diminish noxious stimuli (16, 18). Amplifying nociceptive signals help the animal recognize danger and move away from potential harm. Conversely, reducing these signals can enable the animal to cope during dangerous situations without being overwhelmed by pain. Projection neurons transmit the incoming signal from the dorsal horn to spinal cord pain pathways, connecting this input to various brain structures, such as the brainstem, thalamus, and somatosensory cortex (15, 16). Most nociceptive inputs are transmitted to the thalamus in diencephalon. Serving as a complex yet vital relay center, the thalamus processes sensory signals and redistributes them to the cerebral cortex, where the final conscious perception of pain occurs (15, 16, 19).

### The architecture of the dorsal horn

**Figure 2:**
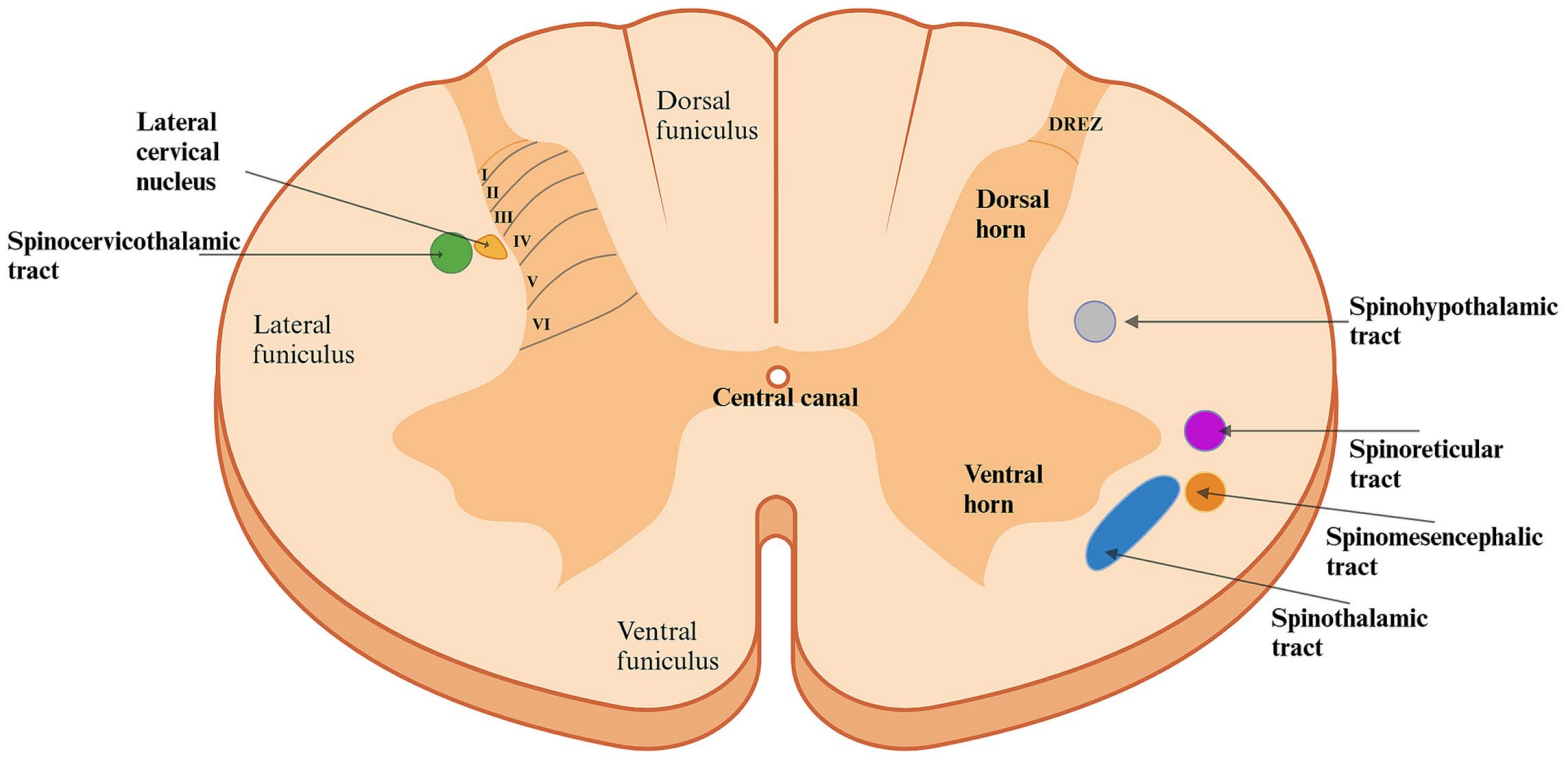
Schematic diagram of the architecture of the spinal cord in the first segments of the cervical region. The dorsal horn is organized into six layers (*laminae*), each with different cell types, compositions, and functions essential for processing and projecting various sensory inputs. Laminae I and II predominantly receive nociceptive input. Moving ventrally through the laminae, the complexity increases: Lamina III primarily receives innocuous (non-nociceptive) inputs and contains a high proportion of interneurons. Besides innocuous input, lamina IV also handles nociceptive input and contains a large proportion of projection neurons. Lamina V, located deeper in the dorsal horn, receives input from deeper structures such as the skin, muscles, and joints, whereas lamina VI is predominantly composed of interneurons and proprioceptive neurons forwarding information. Consequently, nociceptive transmission involves several laminae of the dorsal horn, resulting in various nociceptive pathways, each originating from a different area of the spinal cord, carrying specific input to different areas of the brain. The spinocervicothalamic and spinothalamic tracts are described to be the two most significant nociceptive pathways in the dog and cat. Created in BioRender. Pedersen (2025) https://BioRender.com/l36q898. DREZ: Dorsal Root Entry Zone.

The architectural structure of the dorsal horn was identified by Swedish neurologist Bror Rexed in the 1950s studying spinal cords in cats (20, 21). He described it as being organized into six layers, or laminae (I-VI), each containing different cell types, compositions, and functions crucial for processing and transmitting various types of sensory information (11, 12) (Figure 2). The most dorsal layer, lamina I along with the second layer, the *substantia gelatinosa*, are considered the primary nociceptive regions of the dorsal horn. These layers mainly receive noxious inputs, as well as thermal and touch signals (22). The peripheral afferent fibers can enter the dorsal horn directly through the dorsal horn entry zone or travel up to 2–3 spinal cord segments via the Lissauer’s tract before entering (23, 24). These fibers, originating from dorsal root ganglion neurons can synapse directly onto projection neurons in lamina I or onto dendrites of neurons in deeper laminae that extend dorsally into lamina I (25, 26). Projection neurons from lamina I contribute directly to nociceptive pathways, such as the spinothalamic and spinomesencephalic tracts, while intralaminar neurons in lamina II may connect with other intralaminar neurons or with neurons in other laminae (15, 26–29). The nociceptive tracts will be discussed in further detail in the following section. As we move ventrally through the dorsal horn, the complexity of the laminae’s composition and function increases. Lamina III primarily receives innocuous (non-nociceptive) inputs and contains a high proportion of interneurons, although it also includes a few spinoreticular and spinocervicothalamic projection neurons (28, 30). Similarly, lamina IV receives input from innocuous stimuli but also contains a significant number of projection neurons associated with the spinocervicothalamic tract. As a result, it also responds to noxious stimuli (31–38). Laminae III and IV are collectively referred to as the *nucleus proprius*. Lamina V, located deeper in the dorsal horn, receives input from deeper structures such as the skin, muscles, and joints. It contains a diverse range of neuronal types, including sensory afferent fibers (predominantly C-fibers), wide dynamic range (WDR) neurons, proprioceptors, interneurons, projection neurons, and neurons receiving inputs from the viscera (28). Most of the neurons are multi-receptive and respond to both innocuous and noxious inputs (15, 28). Some participate in the spinothalamic and spinomesencephalic tract (15). The innermost layer of the dorsal horn, lamina VI, consists primarily of interneurons and proprioceptive neurons, along with some spinoreticular projection neurons (28). This lamina is particularly well-developed in the intumescences, reflecting the heightened proprioceptive input from muscles and joints in these regions, but it is less distinct in other areas of the spinal cord (21).

In addition to the gray matter of the spinal cord, the white matter can also be divided into distinct regions. These regions are called *funiculi* and are named according to their locations (Figure 2) (39). The lateral and ventral funiculi consist mainly of axons from projection neurons, including nociceptive tracts (21, 28, 40).

In conclusion, most layers of the dorsal horn receive nociceptive input and play a role in transmitting these signals to the brain.

### Central nociceptive pathways

**Figure 3:**
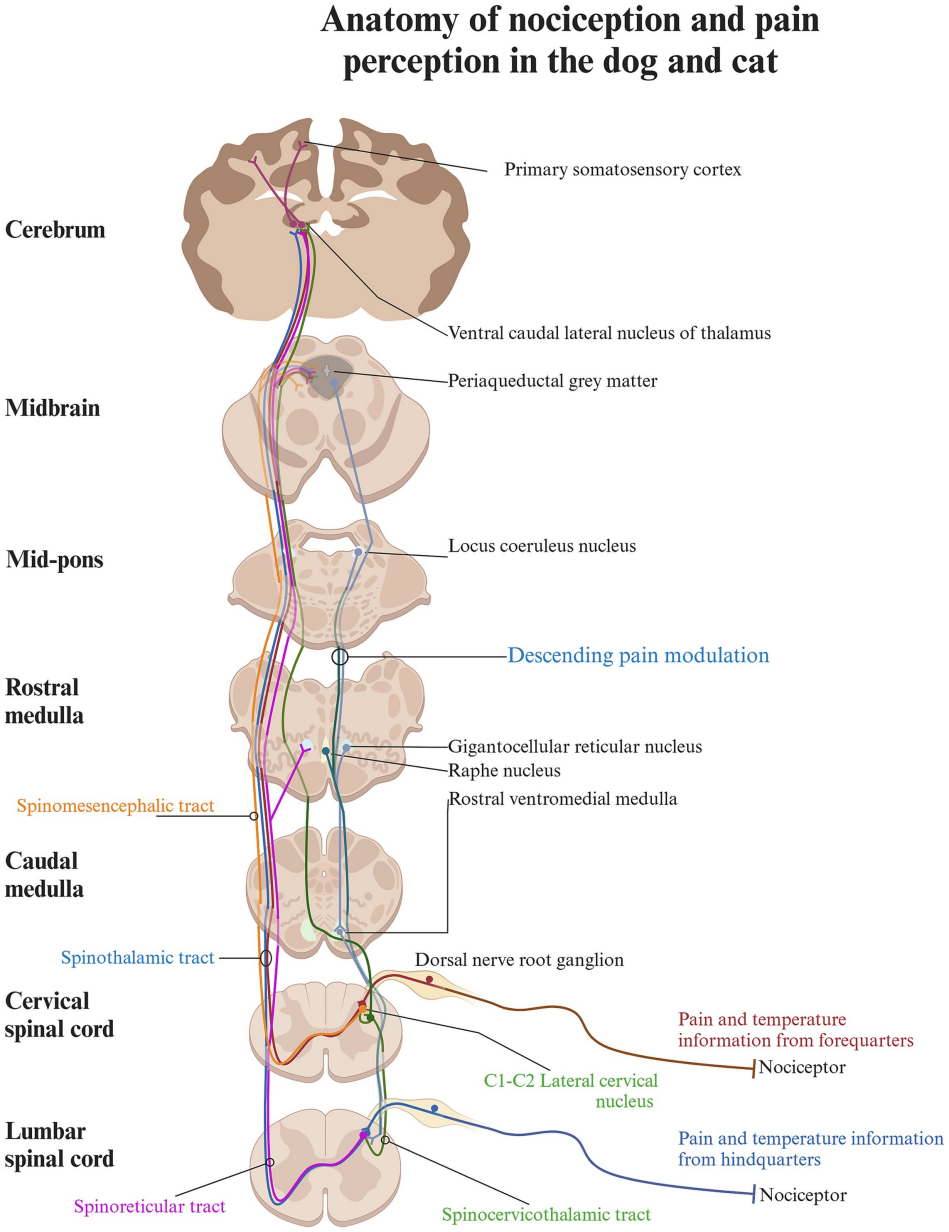
Schematic diagram of the anatomy of nociception and pain perception in the cat and dog. The pathway from registering noxious stimuli to perceiving pain involves multiple nociceptive tracts from various regions of the dorsal horn. Each tract transmits different sensory inputs to specific brain regions, contributing to the perception of pain based on a complex set of input. The spinocervicothalamic and spinothalamic tract are contributing to conscious pain perception, as they relay information to higher brain structures via the thalamus, whereas the spinoreticular and spinomesencephalic tract terminates in the brainstem and the reticular formation and plays a role in the descending modulatory system. Created in BioRender. Pedersen (2025) https://BioRender.com/o86g825.

The path from the detection of noxious stimuli to the perception of pain is not a single, direct route to the brain; rather, it involves multiple nociceptive tracts originating from different regions of the dorsal horn in the spinal cord (Figures 2, 3; Table 1).

**Table 1:** Overview of spinal nociceptive pathways in dogs and cats.

Tract	Origin	Termination	Function	Main neurotransmitters
Spinocervicothalamic tract	Lateral cervical nucleus (located in the white matter outside lamina III-IV in C1 to the cranial part of C3)	Thalamus: Ventral caudal-lateral nucleus	Tactile sensations, nociception	Excitatory: Glutamate Substance P Inhibitory: GABA
Spinothalamic tract	Lamina I + IV-VII	Thalamus: Multiple nuclei including the ventral caudal-lateral nucleus	Nociception, temperature, tactile and mechanical sensations, itch	Excitatory: Glutamate Substance P Inhibitory: GABA Glycine
Spinoreticular tract	Lamina VI-VIII	The reticular formation: the gigantocellular reticular nucleus	Nociception, tactile and mechanical sensations	Excitatory: Glutamate Inhibitory: GABA Glycine
Spinomesencephalic tract	Lam. I, III-V	The reticular formation: various nuclei and the periaqueductal gray	Nociception, mechanical sensations	Excitatory: Glutamate Inhibitory: GABA Glycine
Spinohypothalamic tract	Spinal cord marginal zone, lateral reticulated area and area surrounding the central canal.	Hypothalamus, thalamus, rostral colliculus, reticular formations	Contributes to affective component of pain including reflex autonomic and endocrine responses to painful stimuli and emotional reponse	Excitatory: Glutamate Inhibitory: GABA Glycine

Several spinal nociceptive pathways, with the spinocervicothalamic and spinothalamic tracts being the most prominent, play key roles in pain perception in dogs and cats. Both tracts transmit nociceptive and tactile signals, while the spinothalamic tract additionally conveys sensory information such as temperature and itch. These pathways are integral to conscious pain perception, as they relay information to higher brain structures via the thalamus. In contrast, the spinomesencephalic and spinoreticular tracts terminate in the brainstem, where they contribute to the modulation of pain through the descending inhibitory system.

### The spinocervicothalamic tract

In carnivores, the spinocervicothalamic tract is believed to be the most dominant and biologically relevant nociceptive pathway, followed by the spinothalamic tract (28, 41, 42) (Figure 3). This claim is supported by studies showing that the lateral cervical nucleus, from which the spinocervicothalamic tract projects, is large in carnivores but rudimentary or absent in humans (35, 43). However, there is a lack of robust studies supporting the dominance of the spinocervicothalamic tract in carnivores over other nociceptive pathways, such as the spinothalamic tract. The spinocervicothalamic tract originates from the lateral funiculi of the white matter, just next to laminae III-V across all body regions, projecting input ipsilaterally to the cranial cervical region, where it synapses in the lateral cervical nucleus (20, 38, 44). The lateral cervical nucleus is located in lamina IV between the caudal part of C1 and cranial part of C3 of the spinal cord (38). The neurons of the lateral cervical nucleus receive both innocuous and nociceptive inputs from all parts of the body via the spinocervicothalamic tract. The primary inputs are innocuous tactile signals, which can be triggered by even minor hair movements (31–37). Nociceptive inputs can be triggered by either thermal or mechanical noxious stimuli, such as skin pinching. The receptive fields of these inputs can vary greatly, ranging from very restricted areas (such as a part of a toe) to large or even complete sections of the body (37, 44–46). Conduction velocities vary between 7 and 92 m/s, with glutamate and Substance P serving as key excitatory neurotransmitters, while GABA plays a crucial role in inhibitory neurotransmission (36, 47–53). Signals from the lateral cervical nucleus are transmitted through the dorsolateral funiculus of the spinal cord and ascend through the brainstem, where they decussate to the contralateral medial lemniscus (54–56). These signals primarily terminate in different thalamic nuclei, with the majority ending in the thalamic ventral caudal-lateral nucleus (*nucleus ventralis caudalis*) (54–56). When travelling along the medial lemniscus towards the ventral caudal-lateral nucleus, spinocervicothalamic projecting fibers may branch off and terminate in the midbrain region of the caudal and rostral colliculus (57). In cats, a small percentage of spinocervicothalamic neurons project directly to the periaqueductal gray in the brainstem. The periaqueductal gray is a cell-dense area of gray matter situated centrally around the mesencephalic cerebral aqueduct and plays an important role in descending pain modulation, as well as emotional aspects of pain (58–60). The projection to the periaqueductal gray suggests that the spinocervicothalamic tract may play a role in the descending modulatory pathway or in alerting the animal to potential threats (37, 61, 62).

### The spinothalamic tract

The spinothalamic tract is the most important nociceptive tract in humans, and lesions to it may result in neuropathic pain (63). It is considered an important nociceptive pathway in animals as well. Spinothalamic projection neurons originate from all spinal cord regions and lamina I and IV-VII and transmit with high velocities, suggesting rapid communication of potentially harmful stimuli to higher centers in the body (64, 65). In the cervical spinal cord of cats, spinothalamic cells are primarily somatotopically organized within the dorsal horn, with receptive fields like those of the surrounding interneurons (64). The sizes of spinothalamic receptive fields in the cervical spinal cord are smaller and located to the forelimbs compared to the larger spinothalamic lumbar receptive fields located to the hindlimbs (64, 66). Most spinothalamic neurons traverse the contralateral ventrolateral funiculi of the spinal cord, and a minority travel directly through the dorsolateral funiculi (67–70). Both ascend through the brainstem, close to the medial lemniscus, and terminate in multiple thalamic nuclei (64, 67, 71) (Figure 3). Along their pathway, they send out collateral axonal branches that synapse with various brain structures, including the periaqueductal gray and the reticular formation. This suggests a role in descending nociceptive modulation and alertness behavior (70, 71). The spinothalamic cells receive miscellaneous inputs, ranging from pressure, and temperature to somatic and visceral nociception (64, 69, 70, 72–75). Transmission of stimulation from innocuous mechanical input including light touch and innocuous heat has also been described (64, 70, 74, 75). Specific spinothalamic neurons also respond to histamine and contribute to sensations of itch (68).

Glutamate and Substance P are recognized as key excitatory neurotransmitters of the spinothalamic tract in cats (76, 77). In studies conducted on monkeys, GABA and glycine have been identified as mediators of inhibitory neurotransmission within the spinothalamic tract (78). The role of neurotransmitters is discussed in more detail later in this paper. Studies have shown that spinothalamic cells in cats become sensitized to heat following exposure to intense heat for 30 s (64). Consequently, these cells exhibit a heightened response to lower temperatures afterwards (64).

### The spinomesencephalic tract

The spinomesencephalic tract originates from layers I and III–V in the dorsal horn of the cervical and lumbar spinal cord (15, 29). Its neurons respond to both innocuous and noxious mechanical stimulation from sources such as hair, skin, and deeper structures, including muscles and joints (27, 37, 79). It furthermore receives collateral input from the spinocervicothalamic tract (80).

In rats, glutamate has been identified as a primary excitatory neurotransmitter of the spinomesencephalic tract, while glycine and GABA are suggested to play significant inhibitory roles in cats (81–83). Like the spinothalamic neurons, the spinomesencephalic neurons decussate to the contralateral spinal cord to the ventrolateral funiculi. They ascend through the brainstem, terminating in various nuclei within the reticular formation, including the caudal and rostral colliculi and the periaqueductal gray, with a smaller number continuing to the thalamus (11, 29, 58, 62, 79). Electrical stimulation of the termination sites in the periaqueductal gray has been shown to decrease pain in cats, suggesting that the spinomesencephalic tract may also be involved in descending nociceptive modulation or the emotional aspect of pain (29, 58, 79) (Figure 3). The descending nociceptive modulation as well as the emotional aspect of pain are covered in later sections in greater detail. In cats, spinomesencephalic fibers have furthermore been found to terminate in the parabrachial nucleus, a structure that, in rats, has been shown to contain a significant amount of the endogenous opioid enkephalin (29, 84, 85). Enkephalin, a key pain modulator, binds to opioid receptors throughout the body and is widely distributed in the spinal cord and trigeminal nucleus. In cats, it is particularly concentrated in laminae I, II, and V of the dorsal horn (84–86).

### The spinoreticular tract

The spinoreticular tract primarily originates from the deep layers of the spinal cord (15, 87, 88). Spinoreticular neurons respond to both innocuous and nociceptive stimuli, which range from light touch and hair movement to subcutaneous pressure, as well as lifting and pinching of the skin (17). Glutamate has been identified as a main excitatory neurotransmitter of the spinoreticular tract in cats, while glycine and GABA have been shown to play significant inhibitory roles in rats (89, 90). Spinoreticular neurons also project to the contralateral ventrolateral funiculi, travelling close to the axons from the spinothalamic tract while remaining separate (17). They terminate in the contralateral reticular formation in the brainstem, likely concentrated in the gigantocellular reticular nucleus (17, 87, 91) (Figure 3). Because of their termination in the brainstem, spinoreticular neurons are likely to be involved with both excitatory and inhibitory control of nociception due to activation of the descending modulatory system (17).

### Trigeminal pathway

The spinal tract of the trigeminal nerve is located adjacent to the *fasciculus cuneatus* in the dorsal funiculus (92). It originates from the *nucleus tractus spinalis nervi trigemini* in the brainstem and extends caudally to the dorsolateral funiculi, as well as laminae I and II in the dorsal horn of the first two cervical spinal cord segments (92). This tract plays a crucial role in the transmission of orofacial pain, conveying noxious and sensory information, including touch and temperature, from the face, nasal region, oral cavity, and teeth (92, 93). A more detailed exploration of this pathway lies beyond the scope of this paper.

### Nociceptive mechanisms explained

Nociception occurs when nerve cell fibers are initially activated by a harmful stimulus, which may result from physical contact (mechanical), extreme temperature (thermal), or inflammation (chemical). This process takes place in peripheral organs, including the skin, joints, or muscles as well as internal organs (94). Nociception occurs in four phases: (1) Transduction, (2) Transmission, (3) Modulation, and (4) Perception.

### Transduction phase

The initial stage of nociception is the transduction phase, where nociceptors convert harmful or potentially damaging stimuli into electrical signals. Nociceptors are specialized high-threshold sensory receptors, often described as peripheral free nerve endings. However, recent findings in humans and mice suggest they may not be entirely free but are connected to specialized nociceptive Schwann cells that form mesh-like networks around the nerves and project into the epidermis alongside the nociceptors (95). Such Schwann cells have also been found in the hairy skin of cats (96). Electrical signals are transmitted from the nociceptors by peripheral afferent pseudounipolar neurons located in the dorsal root ganglion outside the spinal cord (9, 10). They are characterized by having split axons that allow them to connect inputs from the periphery to the spinal cord and contain a subset of nociceptive fibers called Aδ or C fibers. These fibers differ in various aspects, such as diameter, degree of myelination, and conduction velocity (9). Aδ fibers are small neuronal fibers (about 2–6 μm in diameter) with a thin layer of myelin, enabling them to transmit signals at high velocity (2, 97). Due to their rapid response, they are responsible for generating “the first pain,” which is a short-lasting, sharp, and pricking sensation that serves as a warning sign (93, 98). Twenty percent of all cutaneous Aδ fibers respond exclusively to noxious mechanical stimulation, while others are also involved in mediating thermal nociception (11, 99). The small surface area and clustered nociceptors of Aδ fibers enable precise identification of sensations, allowing the body to accurately pinpoint the specific area under threat (100, 101). In contrast, the C fibers are smaller (about 0.4–1.2 μm in diameter) and have no myelin, resulting in slower conduction velocities (2, 97). They are responsible for “the second pain,” a throbbing or burning sensation that develops slowly but lasting longer and is difficult to localize precisely due to their large surface areas (93, 98). C fibers can respond to thermal, chemical, and mechanical stimulation and make up 40–90% of the afferent fibers in a cutaneous nerve in cats (15, 99). In general, somatic tissues have a higher density of nociceptors with smaller receptive fields, whereas visceral tissues have fewer nociceptors, but larger receptive fields (11). This often makes it easier to localize somatic pain to a specific area, whereas visceral pain tends to be more diffuse (11). Certain types of C fiber nociceptors can be inactive (silent’) under normal conditions but may become activated in response to specific stimuli. This activation can occur during periods of inflammation, which chemically stimulates the nociceptors. Once activated, these nociceptors can also respond to mechanical or thermal stimulation (11). Besides Aδ and C fibers, peripheral afferent neurons may also contain Aβ fibers. Aβ fibers are large low-threshold neuronal fibers (> 10 μm in diameter) that typically respond to innocuous stimuli and are rarely involved in nociception (2, 97). However, under certain conditions, these fibers may undergo a functional switch, beginning to produce Substance P (93, 102). This change is critical in the development of tactile allodynia, a condition in which normally non-painful mechanical stimulation, such as touch, triggers a painful response (93). This phenomenon can be observed in some patients with neuropathic pain (93).

### Common neurotransmitters and receptors

The generation, transmission, and modulation of nociceptive signals in the dorsal horn of the spinal cord is a complex process involving the release of various neurotransmitters. These neurotransmitters can have excitatory, inhibitory, or dual functions depending on the receptors to which they bind (Table 2) (14). Most neurotransmitters are either amino acids or neuropeptides, and nociceptors can be classified into those that contain neuropeptides and those that do not (103).

**Table 2:** Common neurotransmitters and receptors involved in nociception and pain.

Neurotransmitter	Receptor	Function
Glutamate	AMPA NMDA Kainate	Excitatory
Substance P	Neurokinin 1 (NK1)	Excitatory
CGRP	Neurokinin receptors such as CALCRL	Excitatory
GABA	GABAA GABAB	Inhibitory
Glycine	GlyR	Inhibitory
Serotonin	5-HT	Excitatory/inhibitory
Noradrenaline	α1 α2 β1 β2 β3	Excitatory/inhibitory

Various excitatory and inhibitory neurotransmitters are involved in the generation of pain. Excitatory neurotransmitters such as glutamate bind to α-amino-3-hydroxy-5-methyl-4-isoxazolepropionic acid (AMPA) and N-methyl-D-aspartate (NMDA) receptors, generating rapid action potentials that are critical for sensory processing, whereas Substance P and Calcitonin Gene-Related Peptide (CGRP) bind to Neurokinin 1 (NK1) receptors, generating prolonged postsynaptic potentials to enhance NMDA receptor activity. Inhibitory neurotransmitters, including GABA and glycine, modulate sensory inputs and helps maintain a balance between excitatory and inhibitory activity in the central nervous system. AMPA: α-amino-3-hydroxy-5-methyl-4-isoxazolepropionic acid; CALCRL: Calcitonin- receptor-like receptor; CGRP: Calcitonin Gene-Related Peptide, NK1: Neurokinin 1; NMDA: N-methyl-D-aspartate.

Excitatory neurotransmitters, such as glutamate - the primary excitatory neurotransmitter in the central nervous system - are important for generating fast, short-lived signaling throughout the nervous system including the thalamocortical areas (15). Glutamate binds to the *α*-amino-3-hydroxy-5-methyl-4-isoxazolepropionic acid (AMPA) and N-methyl-D-aspartate (NMDA) receptors, generating rapid action potentials that are critical for sensory processing, including nociceptive pathways (11, 14, 104). Neuropeptides, including Substance P and Calcitonin Gene-Related Peptide (CGRP), bind to neurokinin receptors, such as the neurokinin 1 (NK1) receptor, to generate slower, prolonged postsynaptic potentials which may reinforce the effect of the activated AMPA receptors (11). Primary afferent C-fibers release both excitatory neuropeptides and amino acids including Substance P and glutamate (105, 106). By producing these mediators, along with cytokines and other chemical signals during a painful condition, additional inflammatory cells are recruited and may participate in prolonging the sensation of pain (93).

In the dorsal horn, Substance P facilitates nociceptive transmission by binding to NK1 receptors located primarily in lamina I-III (106, 107). Other peptides, such as CGRP, can further enhance NMDA receptor activity, inducing a sensitized state that increases nociceptor sensitivity. This heightened sensitivity may contribute to the development and persistence of chronic pain conditions (16). Calcitonin Gene-Related Peptide is found in Aδ and C fibers within the dorsal root ganglion, Lissauer’s tract, and the terminals of primary afferent fibers, particularly in laminae I and II (108). Alongside excitatory neurotransmitters, inhibitory neurotransmitters like GABA and glycine play a crucial role in modulating incoming sensory inputs. GABA is the primary inhibitory neurotransmitter in nociceptive processes, playing a crucial role in maintaining the balance between excitatory and inhibitory activity within the central nervous system (16, 105). In the dorsal horn, GABA receptors are widely distributed including on primary afferent terminals, interneurons and descending fibers (109, 110).

Serotonin and noradrenaline are involved in both inhibitory and excitatory processes. Serotonin primarily originates from the raphe nuclei in the brainstem, while noradrenaline is primarily sourced from the locus coeruleus in the brainstem (16). These neurotransmitters are integral components of the descending modulatory system, which regulates nociceptive processing (111–114). Serotonin and noradrenaline may also be released during normal physiological events or in response to inflammation, where they participate in lowering the nociceptive threshold (15, 104). The role of serotonin and noradrenaline as part of the descending modulatory system is discussed later in this paper.

The principle behind pain management is based on pharmacological modulation of neurotransmitters and receptors. Drugs such as gabapentin and pregabalin modulate voltage-gated calcium channels, reducing the release of glutamate and Substance P (115, 116). Maropitant, a drug that blocks the binding of Substance P to its receptor (NK1), may offer a potential treatment option for neuropathic pain although it is primarily used as an antiemetic in veterinary practice. Its analgesic effects in humans have been largely disappointing, though it has shown some variability in analgesic responses in cats and can be used to reduce phantom scratching in dogs with syringomyelia (117, 118). Tramadol, a synthetic analgesic, is metabolized in the liver into two metabolites, one acts as an opioid agonist, and the other enhances the inhibitory effects of noradrenaline and serotonin (117). Amitriptyline, a tricyclic antidepressant, is occasionally used to treat chronic neuropathic pain in animals. It has a complex pharmacological profile with actions on serotoninergic and noradrenergic systems as well as anticholinergic and antihistaminergic properties and NMDA receptor antagonism (117). Selective serotonin-norepinephrine reuptake inhibitors (SSRIs), another class of antidepressants, work by inhibiting the reuptake of serotonin and noradrenaline, thereby prolonging their effect, and potentially alleviating neuropathic pain (117).

Magnesium has been investigated as a potential therapeutic agent for pain management due to its antagonistic action at NMDA receptors, preventing the process of central sensitization and mitigating preexisting pain hypersensitivity (119). Central sensitization is discussed in greater detail in the section on modulation. As a natural calcium antagonist, magnesium may also exert its analgesic effects through calcium channel blockade, which has been identified as a therapeutic target in neuropathic pain conditions (119). In combination with NMDA antagonists such as ketamine, magnesium has been shown to enhance the drug’s binding affinity and inhibit calcium influx through blockade of the NMDA receptor, suggesting a synergistic effect in pain modulation (119).

Preventative strategies may also be beneficial in reducing or preventing central sensitization. Local anaesthetic blocks can help prevent the induction of central sensitization in animals with pre-existing painful conditions undergoing procedures, such as cats with feline orofacial pain syndrome or tooth resorptions undergoing dental care (100). By incorporating targeted interventions, the progression of central sensitization may be attenuated, thereby improving pain management outcomes and chronification of pain.

### Transmission phase

During the transmission phase, nociceptive signals are transported from the periphery through the spinal cord and to the brain. When peripheral afferent neurons reach the dorsal horn, the primary area for somatosensory processing in the spinal cord, they synapse with projection neurons. Projection neurons have long axons that can extend across multiple spinal cord segments and brain regions before finally terminating in the brain (57). Projection neurons may also have collateral branches that synapse in various regions along their pathway (120, 121). Depending on the region of the dorsal horn from which they originate, projection neurons are characterized by specific neuroanatomical features, such as termination site, axon length, number of collateral branches, and the nociceptive input they transmit. In the spinal cord, nociceptive projection neurons may be activated only by nociception (nociceptive-specific), or they may respond to a range of stimuli, including light touch and different ranges of noxious stimulation (WDR neurons) (62, 63).

Nociceptive-specific neurons are typically found in the superficial laminae of the dorsal horn, while WDR neurons are located in deeper layers. However, WDR neurons may contain large dendrites that extend into the superficial layers to synapse with primary afferent fibers (98, 122).

### Modulation

In 1965, Melzack and Wall introduced the gate control theory of pain to explain phenomena such as the relief of itching or pain sensation when rubbing a nearby area (107, 123). They proposed that lamina II in the dorsal horn acts as a “gate,” which opens and closes to sensory and nociceptive peripheral inputs before projecting them to the brain. Essentially, this theory describes how the activation of Aβ fibers by non-noxious stimuli can disrupt the transmission of nociceptive signals by C fibers (99, 100). Although later studies have found the gate control theory limited due to the diversity of neurons involved, it significantly advanced our understanding of pain physiology and nociceptive modulation (124, 125).

Nociceptive signals undergo numerous complex modulations before reaching the brain and being perceived as pain (15, 19). Modulation designates the process in which descending projection neurons, interneurons or the brain interact with incoming noxious input in the spinal cord, enhancing or reducing the signal (15, 19, 126). This modulation involves different adaptations and reorganization in response to injury, persistent nociceptive input, or external stimuli and is referred to as nervous system plasticity or ‘neuroplasticity’ (19). This phenomenon occurs throughout the nervous system, including the peripheral nerves, where nociceptors may become sensitized (peripheral sensitization), the spinal cord (central sensitization), and the brain (via altered processing in pain-related cortical regions or through changes of the descending pathways) (19, 127). These alterations result from molecular changes, such as the upregulation of excitatory neurotransmitters like glutamate and the downregulation of inhibitory mediators such as GABA (19).

Spinal and supraspinal mechanisms play crucial roles in controlling nociceptive inputs to the brain (125).

### Spinal modulation

In the spinal cord, interneurons play a crucial role in local modulation by directly or indirectly influencing projection neurons through the release of various neurotransmitters (126). Nitric oxide (NO) is a key signaling molecule involved in the modulation of nociception within the spinal cord. Its production is primarily triggered by calcium influx through NMDA receptors, which activates nitric oxide synthase (NOS). However, NO can also be generated in response to tissue damage and inflammation, as pro-nociceptive mediators—including hydrogen ions (H^+^), potassium ions (K^+^), and adenosine triphosphate (ATP)—activate NOS (14, 128–130). The involvement of NO in nociception is particularly prominent in inflammatory and neuropathic pain (14, 129). Inflammatory states lead to the release of cytokines and prostaglandins, which can induce NO production in certain neurons and glial cells within the dorsal horn of the spinal cord (128, 131). NO facilitates pronociceptive signaling by sensitizing dorsal horn neurons, enhancing excitatory neurotransmitter release from primary afferent nociceptive fibers, and promoting the synthesis of prostaglandins and cytokines (128).

### Wind-up and central sensitization

Pain perception is significantly influenced by spinal cord and medullary dorsal horn neurons through a process known as “wind-up.” This phenomenon is characterized by a progressive and prolonged increase in neuronal excitability following repetitive stimulation of C fibers at a constant intensity. It involves neurotransmitters such as Substance P and glutamate, which act on their respective receptors, NK1 and NMDA (132, 133). Under normal conditions, NMDA receptor channels are blocked by magnesium ions, preventing calcium influx despite glutamate binding (119, 134). However, depolarization of the post-synaptic membrane—either through Substance P activation of NK1 receptors or glutamate binding to AMPA receptors—removes this magnesium block, allowing NMDA receptor activation (119, 134). Repeated NMDA receptor activation, along with increased glutamate influx and voltage-gated calcium channel activity, triggers intracellular signaling cascades that progressively enhance neuronal firing frequency and intensity, even with low-frequency stimulation (119, 134). This process increases synaptic plasticity and enhances dorsal horn neuron excitability, making them more responsive to incoming pain signals (130, 134). Wind-up is a critical early step in the development of central sensitization.

Central sensitization is driven by key neurotransmitters, including glutamate, Substance P, and brain-derived neurotrophic factor (BDNF), which interact with receptors such as NMDA, mGluR, NK-1, and TrkB (130). These interactions elevate intracellular calcium levels and activate intracellular signaling pathways, leading to enhanced receptor function and increased receptor recruitment to the cell surface, ultimately amplifying synaptic reactivity (130). Other mechanisms contributing to central sensitization include ectopic action potential generation, facilitation and disinhibition of synaptic transmission, immune cell interactions, and structural synaptic changes (100). The result is an amplification of pain signals and heightened pain perception, which can persist long after the resolution of the initial injury or inflammation (100).

### Glial cells

Another important spinal modulator are the glial cells. Glial cells, especially astrocytes and microglia, play important roles in the development and persistence of chronic and neuropathic pain by actively modulating neuronal activity and contributing to neuroinflammation (135–137). Microglia influences plasticity and synaptic function through neuronal interactions (138). When activated, microglia can release pro-inflammatory cytokines and chemokines, promoting neuroinflammation that contribute to sustain chronic pain conditions (139).

Astrocytes are the most abundant glial cells in the central nervous system, where they support homeostasis by maintaining synaptic function, regulating and recycling neurotransmitters, and releasing other neuromodulatory substances such as growth factors (139, 140).

The activation of astrocytes and microglia in sensory pathways can enhance nociceptive signaling by increasing neuronal excitability and reduce the body’s inhibitory control, which potentially may lead to increased and abnormal pain sensations through central sensitization as seen in chronic and neuropathic pain conditions (125, 141). This can occur with dorsal horn nerve injuries, where microglial activation triggers the synthesis and release of BDNF (125, 142). BDNF reduces GABAergic inhibition, which paradoxically can result in excitatory effects – a phenomenon called disinhibition, which leads to further sensitization and neuroplastic changes in the pathway (125, 142). Pathological activation of astrocytes can exacerbate neuroinflammation, inhibit axonal growth, and suppress synapse formation, thereby disrupting sensory neural circuitry (136).

In the dorsal root ganglion, satellite glial cells amplify nociceptive signals and sustain inflammation by releasing growth factors and chemokines that attract immune cells (143).

### Supraspinal modulation

Nociceptive signals are also modulated by supraspinal structures. The descending modulatory system, which regulates nociception at the level of the spinal dorsal horn, involves three key components: the rostral ventromedial medulla (an important source of the neurotransmitter serotonin), located on the floor of the medulla oblongata; the periaqueductal gray, a cluster of neuronal cell bodies surrounding the mesencephalic cerebral aqueduct (containing multiple neurotransmitters including glutamate, GABA, opioids particularly enkephalin, Substance P, neurotensin and endocannabinoids); and the nucleus locus coeruleus, situated in the pons (source of noradrenergic neurotransmitters) (15). These structures influence spinal projection neurons either pre- or postsynaptically, thereby modulating the transmission of nociceptive signals to the brain (18). Studies have shown that up to 50% of projection neurons in laminae I and V of the lumbar spinal cord interact directly with enkephalin terminals, an endogenous opioid with pain-suppressing effects suggesting the presence of a post-synaptic endogenous pain regulation mechanism in the feline spinal cord (85, 99). This is most likely also the case in dogs, where the distribution of enkephalins in the brain is largely similar to cats (144).

*The periaqueductal gray* receives input from both higher and lower structures within the central nervous system and can respond by releasing a variety of endogenous opioids, including enkephalin to modulate the inputs (59, 144–146). Additionally, the periaqueductal gray can activate other components of the descending modulatory system, such as the raphe nuclei located in the rostral ventromedial medulla (11, 114). The raphe nuclei are a critical source of serotonin and play a role in serotonergic modulation within the spinal cord (11). The analgesic effect from activation of the raphe nuclei has also been shown to involve enkephalin (147). Furthermore, the raphe nuclei can stimulate the locus coeruleus, which is the primary source of noradrenaline in the body. Noradrenaline binds to α2-receptors in the spinal cord, producing an analgesic effect (15). This mechanism is mimicked when α2 agonists are used during anaesthesia (15). Both serotonin and noradrenaline inhibit the transmission of ascending nociceptive signals from the spinal cord by activating interneurons and blocking nociceptive inputs from all layers of the dorsal horn (111–114). In mice, nociceptive neurons in lamina V-VIII project back to nucleus raphe magnus, which is part of the raphe nuclei complex. This establishes a nociceptive processing-loop between the deeper dorsal horn and the raphe nuclei (148). In cats, serotonin binding follows a laminar distribution in the spinal cord, with high binding levels in laminae II and III and reduced binding in the deeper dorsal horn laminae, except for lamina I, which also shows low binding (113). Dogs exhibit a similar pattern, where the density of serotonin fibers decreases ventrally through the dorsal horn, with low-level expression in lamina II (149). The clinical effect of this species-specific difference is unknown to the authors. Additionally, the thoracolumbar spinal cord is richer in serotonin fibers than the cervical cord (149). These serotonin fibers travel craniocaudally through the white matter funiculi, sending horizontal branches into the gray matter and extending to the tail and *filum terminale* (149).

In human chronic neuropathic pain conditions, the phenotype of the descending inhibitory pathway may shift from being predominantly controlled by noradrenergic and serotonergic mechanisms to involving other neurotransmitters (63). Additionally, this pathway can become depleted, leading to reduced opioid receptor function (93). Since opioids play a crucial role in the descending inhibitory pathway, this impairment results in increased neuronal firing at the spinal cord level, ultimately amplifying pain perception (93).

### The role of the thalamus in nociceptive transmission and pain generation

**Figure 4:**
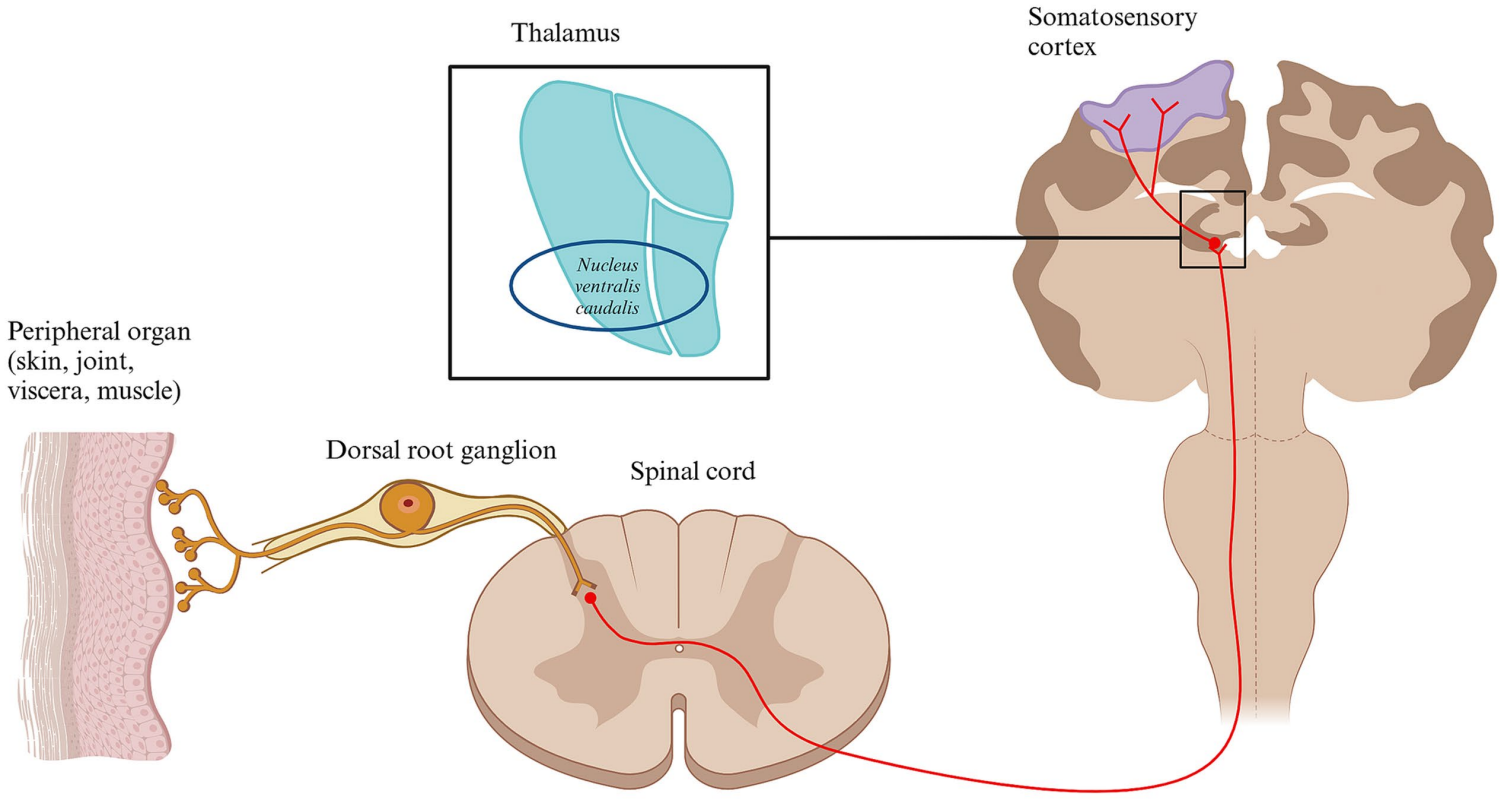
Schematic diagram of the thalamus and the somatosensory cortex. Conscious pain perception is caused by the activation of several brain structures. Following transmission through different nociceptive pathways, most nociceptive inputs reach the thalamus. One of the most important thalamic nucleus for processing nociceptive input is the ventral caudal-lateral nucleus. From here, the nociceptive signals are distributed to multiple higher brain areas, including the primary and secondary somatosensory cortex, contributing to the subjective perception of pain. Other relevant brain structures involve amygdala, the insula and the cingulate cortex. Created in BioRender. Pedersen (2025) https://BioRender.com/t93x590.

Before reaching higher brain structures and being perceived as pain, most nociceptive signals pass through the thalamus. The thalamus is a gray matter structure composed of several nuclei located in diencephalon that processes and distributes most of the sensory inputs coming from the body or higher brain structures. The thalamus in cats and dogs is quite similar and contains 18 nuclei grouped together (150). It is divided into external and internal laminae, which are further organized based on their anatomical orientation (92, 150). The intralaminar nuclei, which are activated by axons from the reticular formation, are grouped into medial and lateral categories (92, 150). When activated from nociceptive inputs, the medial thalamic nuclei help generate emotional and motivational aspects of pain due to its connection to the limbic system (15). In contrast, lateral nuclei are involved in the sensory-discriminative aspects of pain, projecting to the somatosensory cortex (15). Both groups are further divided into dorsocaudal and ventral nuclei (92). The ventral nuclei are key for transmitting nociceptive signals and include subgroups like ventral rostral, lateral, caudal, and medial nuclei (92, 150). Specifically, the ventral caudal-lateral nucleus (*nucleus ventralis caudalis*) relays pain signals to the somatosensory cortex (92) (Figure 4).

### Perception

Conscious pain perception arises from the activation of multiple brain structures collectively referred to as the pain matrix. This term describes a theoretical cortical network involved in pain perception, integration, and modulation. The pain matrix includes the amygdala, hippocampus, somatosensory cortex, insular cortex, cingulate cortex, prefrontal cortex, and regions within the frontal and parietal lobes (100).

This network integrates emotional and cognitive processes, generating both emotional and behavioral responses to pain.

Pain perception within the pain matrix can be categorized into three main components:

1. Sensory-discriminative – responsible for the localization, intensity, and duration of pain.
2. Affective-motivational – related to the emotional response to pain.
3. Cognitive – governing the behavioral and evaluative aspects of pain

The primary and secondary somatosensory cortices (S1 and S2) play a crucial role in the sensory-discriminative aspect of pain, encoding information about its location, intensity, and duration (100).

However, pain perception is influenced not only by the sensory registration of nociceptive signals but also by various other factors, including environment, past experiences, cognitive function, and emotional state (100, 151). Just as lower components of the pain pathway undergo modulation, the brain itself can also adapt, potentially altering pain perception (152). Central sensitization within the dorsal horn can amplify and enhance nociceptive inputs reaching the brain, intensifying pain perception (19). Under normal conditions, neuroplasticity supports healing and adaptation. However, in chronic pain conditions, prolonged exposure to heightened nociceptive input can lead to maladaptive changes in brain regions associated with pain processing (6, 153, 154).

These changes often involve increased activity in areas linked to the emotional and cognitive aspects of pain, such as the rostral (anterior) cingulate cortex and insula (154, 155). This highlights the importance of a comprehensive pain management approach, particularly when conventional treatments fail to achieve the desired outcomes. Effective pain management should address not only the sensory aspects of pain but also the maladaptive neural changes and the cognitive and emotional factors that shape the pain experience.

### Cognitive component of pain

The cranial cingulate cortex, in addition to the prefrontal cortex, plays a role in advanced cognitive behavior, decision-making, and expression of both personality and social behavior (100). In cats, a direct projection from the ventral area of the thalamus, including the ventral caudal-lateral nucleus, to the cingulate cortex has been identified, providing a direct connection between nociceptive inputs and cognitive behavior (156). A recent study found decreased volume of the cingulate gyrus in Cavalier King Charles Spaniels with Chiari-like malformations and syringomyelia, supporting the hypothesis that chronic pain can also change the overall function of the central nervous system in the dog brain (153). Pain-induced changes to the cingulate cortex may therefore affect cognitive or emotional functions. This has recently been demonstrated in a study reporting that chronic musculoskeletal pain in working dogs may affect cognitive function by impairing spatial working memory (157).

### Emotional component of pain

**Figure 5:**
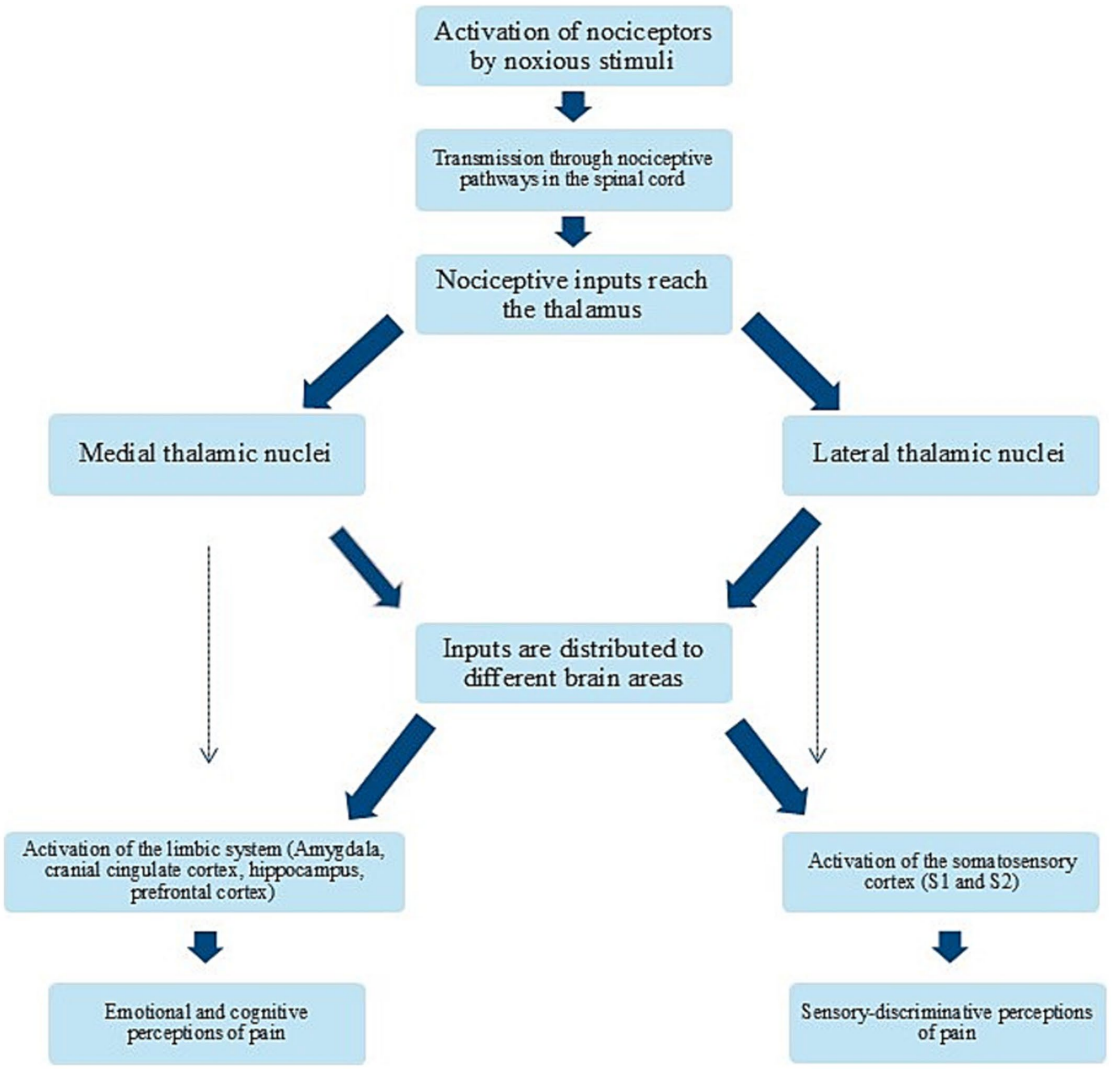
Flow chart of the process from nociception to pain perception. Simplified flow chart illustrating the process from nociceptor activation to conscious perception of pain and its consequences.

Although pain-related effects on emotions have not been well studied in animals, they are well documented in humans with chronic pain. People suffering from chronic pain often experience depression and anxiety along with higher degrees of frustration (158). This is believed to arise from an increased activation of the limbic system by nociceptive input. The limbic system contains regions involved in emotions, feelings, and memory. In pain perception, the limbic system helps generate memories and behaviors that serve to protect and prevent the individual from a future painful situation. In particular the amygdala and hippocampus are important for the generation and consolidation of pain-related memories, serving the purpose of preventing the animal from encountering a similar situation in the future. However, together with the prefrontal cortex the amygdala and hippocampus can contribute to a negative emotional state and anxiety triggering the release of adrenaline following a painful stimulation (100). Similarly, the periaqueductal gray not only processes nociceptive information, but also processes other sensory inputs that are relevant for regulating emotional motor behaviors, including defensive responses such as fighting or flight (59, 60, 159). The lateral periaqueductal gray mediates autonomic hypertension, whereas the caudolateral region is associated with flight behavior. Studies in cats have shown that spinocervicothalamic and spinomesencephalic neurons project to these areas, suggesting that nociception mediated by these nociceptive tracts may mediate hypertension and behavior associated with anxiety (60–62). Nociceptive-induced activation of these brain structures creates a strong relationship among negative emotional states, anxiety, and pain that should not be forgotten in animals. A recent study showed that fear and anxiety, as well as the dog’s ability to handle novel or challenging situations and engage socially, may be predictors of chronic pain (160). Dogs with chronic musculoskeletal pain had a higher frequency of fear and aggression and had poorer coping ability with daily changes (160). Similarly, chronic stress, such as social conflict, can exacerbate pain conditions, as observed in cats with feline orofacial pain syndrome. This highlights a bidirectional relationship between stress, anxiety, and pain, where stress amplifies pain perception, and persistent pain, in turn, increases stress and anxiety (100). Thus, the synchronous activation of multiple brainstem and higher brain structures are contributing to the full pain experience (Figure 5). It is therefore important to consider pain not only a sensory-discriminative experience, but also an emotional experience and promote positive emotions as a multimodal component when treating veterinary patients with pain.

### Limitations of this review

The field of pain research is vast, encompassing diverse topics from studies on ion channels and intracellular structures to investigations using rodent models and clinical studies in large mammals, primarily humans. This review aims to provide a broad overview tailored for veterinary clinicians and does not attempt to be exhaustive. Readers seeking more detailed or comprehensive insights are encouraged to consult specialized journals and reviews dedicated exclusively to pain research.

## Conclusion

A thorough understanding of pain processes and pathways is essential for veterinary professionals to deliver effective pain management for canine and feline patients. This review highlights key aspects of spinal nociception and pain in dogs and cats. While significant advancements have been made in understanding nociception and pain pathways in these species, further research is needed, particularly on biomarkers of chronic pain, methods of assessing and monitoring animals with chronic pain and species-specific mechanisms of cognitive and emotional pain perception in chronic pain conditions.
